# Frequency of Thyroid Dysfunctions during Interferon Alpha Treatment of Single and Combination Therapy in Hepatitis C Virus-Infected Patients: A Systematic Review Based Analysis

**DOI:** 10.1371/journal.pone.0055364

**Published:** 2013-02-01

**Authors:** Chandrasekharan Nair Kesavachandran, Frank Haamann, Albert Nienhaus

**Affiliations:** 1 Centre for Epidemiology and Health Services Research in the Nursing Profession (CV Care), University Medical Centre Hamburg-Eppendorf, Hamburg, Germany; 2 Institute for Statutory Accident Insurance and Prevention in the Health and Welfare Services, Department of Occupational Health Research, Hamburg, Germany; 3 Epidemiology Division, CSIR-Indian Institute of Toxicology Research, Lucknow, India; Centro di Riferimento Oncologico, IRCCS National Cancer Institute, Italy

## Abstract

**Background:**

Thyroid dysfunction is the commonest endocrinopathy associated with HCV infection due to interferon-based treatment. This comprehensive and systematic review presents the available evidence for newly developed thyroid antibodies and dysfunctions during interferon treatment (both single and combination) in HCV patients.

**Methodology/Principal Findings:**

This systematic review was conducted in accordance with the PRISMA guidelines. The data generated were used to analyze the risk for thyroid dysfunctions during interferon (IFN) treatment in HCV patients. There was a wide range in the incidence of newly developed thyroid dysfunctions and thyroid antibodies in HCV patients during IFN treatment (both single and combination). The wide range of incidence also denoted the possibility of factors other than IFN treatment for thyroid-related abnormalities in HCV patients. These other factors include HCV viral factors, genetic predisposition, environmental factors, and patho-physiological factors. Variations in IFN dosage, treatment duration of IFN, definition/criteria followed in each study for thyroid dysfunction and irregular thyroid function testing during treatment in different studies influence the outcome of the single studies and jeopardise the validity of a pooled risk estimate of side effects of thyroid dysfunction. Importantly, reports differ as to whether the thyroid-related side effects disappear totally after withdrawal of the IFN treatment.

**Conclusions/Significance:**

The present review shows that there is a wide range in the incidence of newly developed thyroid dysfunctions and thyroid antibodies in IFN treated HCV patients. This is a comprehensive attempt to collate relevant data from 56 publications across several nations about IFN (both mono and combination therapy) related thyroid dysfunction among HCV patients. The role of each factor in causing thyroid dysfunctions in HCV patients treated with IFN should be analyzed in detail in future studies, for a better understanding of the problem and sounder clinical management of the disease.

## Introduction

As per the World Health Organization (WHO), nearly 3% of the global population suffers from Hepatitis C Virus (HCV) infection, prevalence of the same ranging from 0.1–5% is reported for different European countries [1], [2]. Interferon alpha (IFN α) - singly and in combination with other drugs - has been popularly used to treat the HCV infection [3], [4]. However, despite its success, this treatment causes several side effects in the HCV patients, including influenza-like symptoms, hematological effects, neuropsychiatric symptoms and, significantly, various thyroid-associated diseases [5]. Severe and even life-threatening side effects of IFN reportedly occur in 0.1 to 1% of patients treated; these include thyroid, visual, auditory, renal and cardiac impairment and pulmonary interstitial fibrosis [6], [7].

A higher prevalence of thyroid disorders has been reported in HCV-infected patients than in the general population [8]. Indeed, thyroid dysfunction is the most common endocrinopathy associated with the IFN-based treatment of HCV infection [7]. Interferon-induced thyroiditis (IIT) is a major clinical problem for patients who receive IFN therapy, with complications like thyrotoxicosis being especially severe [9], [10], [11], [12]. Thyroid diseases have been reported due to treatment based on IFN α as well as IFN ß [4].

IFN has important immunomodulatory properties due to which it can induce autoimmune phenomena like autoimmune thyroiditis with hypo - or hyperthyroidism [8]. Autoimmune thyroiditis has been reported in up to 20% of the patients during IFN-based therapies in a review article [13]. Thyroid dysfunction may also manifest as destructive thyrotoxicosis, Graves’ thyrotoxicosis and hypothyroidism. These pathological conditions may occur in the same patient as a result of different immunological effects of IFNα therapy on the thyroid gland [14]. IFN treatment may also induce a subtle defect in the thyroidal organification of iodide, thus further impairing hormone synthesis [9].

A common drug used with IFN α in HCV treatment is Ribavirin (RIBA) [15]. RIBA is a synthetic analog of guanoside that induces the Th1 cytokines in the immune response against HCV infection [15].When undergoing treatment, IFN and ribavirin synergize to stimulate the immune system in order to eradicate the virus [7]. One innocent bystander in this accentuated response is the thyroid [7].

Such is the correlation between the therapy and the gland malfunction that clinicians have often reduced the dose or sometimes even discontinued IFN α treatment in patients who develop thyroid dysfunction, thus possibly compromising the therapeutic response [16]. The current state of art treatment for HCV patients is a combination of pegylated IFN alpha (2a or 2b) and Ribavirin.

This background, a comprehensive and systematic review presenting the available evidence for the newly developed thyroid antibodies (Tab) and dysfunctions during interferon treatment (both single and combination) in HCV patients was conceived. We have included herein 19591 case studies/patient histories (16149 from mono-therapy and 3442 from combination therapy) from 56 publications (31 mono and 25 combination treatments) to understand the frequency of risk associated with thyroid dysfunctions during IFN treatment (single and combination) among HCV patients.

To the best of our knowledge, this systematic review has included the highest number of case studies and publications to analyze the risk of thyroid dysfunction in patients during both single and combination IFN α treatment compared to earlier studies that were based either on single or combination therapy of IFN α or dealt with limited numbers of patients and publications in earlier narrative and systematic reviews with meta-analysis [7], [17], [18], [19], [20]. The study also analyzes the pre-disposing factors that may cause thyroid dysfunctions in HCV patients.

## Methods

### Search Strategy and Screening

**Figure 1:**
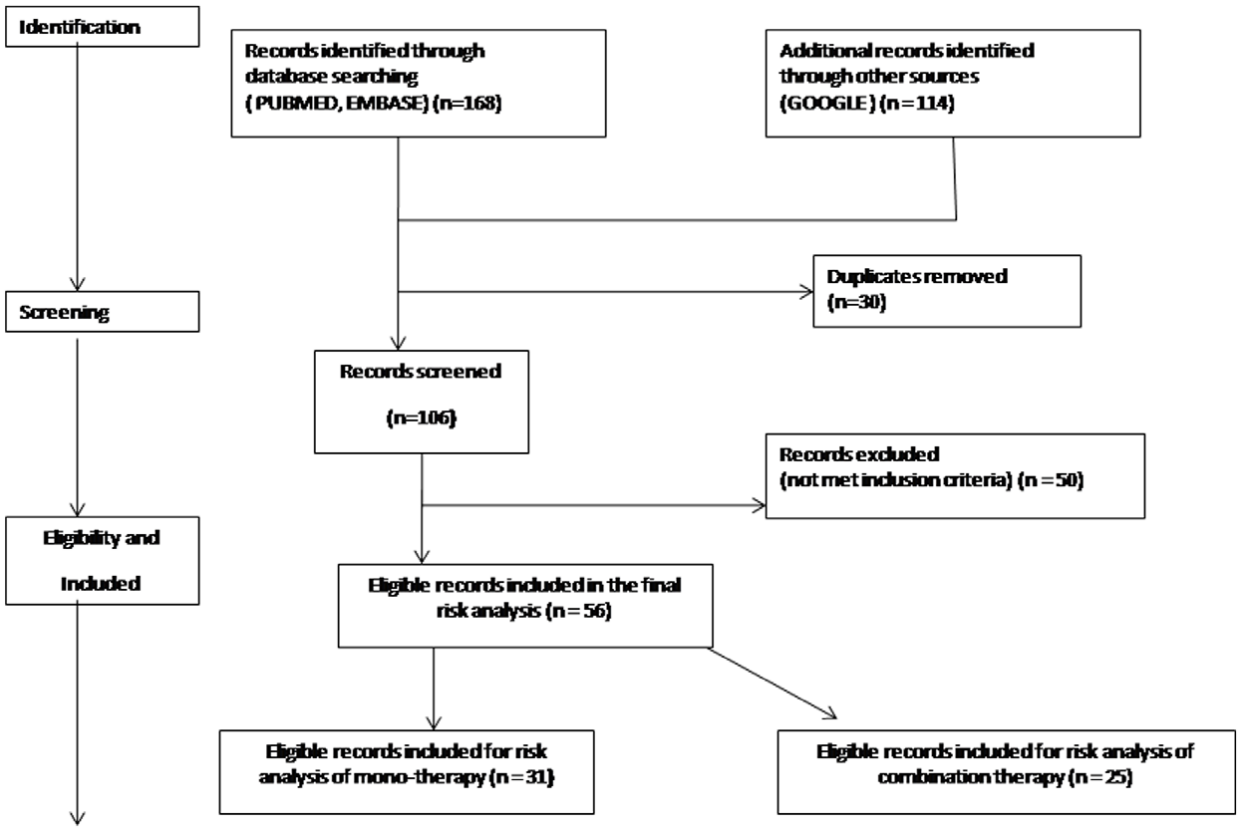
Identification, screening, eligibility and inclusion of data sources for the study.

A systematic literature search was performed using PubMed, EMBASE and Google. The keywords used were ‘interferon treatment’ combined with ‘thyroid’, ‘hepatitis C’, ‘antibodies’, ‘autoimmunity’, ‘dysfunctions’, ‘pegylated’, ‘meta-analysis’, ‘pathogenesis’, ‘molecular mimicry’, ‘genetic predisposition’, ‘Levovirin’, ‘consensus Interferon’, ‘diagnosis’, ‘management’ and ‘ribavirin’ for the period between January 1990 to November 2012. Identification, screening, eligibility and inclusion of database for the study have been depicted in a flow chart (Fig. 1). The flow chart was developed on the basis of the Preferred Reporting Items for Systematic Reviews and Meta-Analyses (PRISMA) for reporting databases in systematic reviews [21]. The systematic review protocol for PRISMA was based on the information available at http://www.prisma-statement.org/statement.htm.

The inclusion of publications for the present study was based on the following criteria:

1. Design of study: Case-control, prospective, retrospective
2. Availability of data on thyroid disease ie., newly developed thyroid dysfunctions (hyper and hypothyroidism),newly developed thyroid antibody (Tab’s) during IFN treatment
3. Treatment must include at least one of the following therapy regimes:

- IFN α
- IFN α+RIBA
- IFN in combination with RIBA [IFNα 2b+RIBA]
- Consensus Interferon-1 (IFN α Con-1)+RIBA
- Pegylated IFN (PEG-IFN) α+RIBA
- Pegylated IFN (PEG-IFN) α+Levovirin
- Consensus Interferon (CIFN) α

German and English Language articles were screened for the study.

### Study Quality

The methodological quality of the literature was assessed as “moderate” or “good”. A study was deemed to be of “moderate” quality if it did not include any of the key words given for search and did not follow the inclusion criteria of publication mentioned above. A study was rated as “good” if publications were relevant to the topic, any two of the above keywords were mentioned in the publication, and it followed the inclusion criteria discussed previously. Only the “good” quality publications were selected for the study.

## Results

### Frequency of Occurrence of Thyroid-related Side Effects among HCV Patients Undergoing IFNα Treatment (Mono and Combination Therapy)

The study found 168 publications from PUBMED and EMBASE and 114 documents from other sources like Google during the systematic database search. Of these, 56 publications were synthesized on the basis of the inclusion criteria and PRISMA guidelines (Fig 1). Table 1 reports the frequency of newly developed thyroid antibodies and thyroid dysfunctions in HCV patients from 31 previous studies, with single and 25 studies pertaining to combination IFN therapy (Table 2). 16149 patients (mono-therapy) and 3442 patients (combination therapy) from different case studies and patient histories were included in the risk analysis from 31and 25 studies from mono-therapy and combination therapy respectively.

**Table 1:** Frequency of newly developed thyroid antibodies and clinical thyroid Disease (Including Autoimmune IIT and Non-Autoimmune IIT) in Patients with Hepatitis C Infection treated with mono therapy (IFN α, Ribavirin) treatment.

Si No	Country	Treatment	No.(M/F)	Newly developed thyroid antibody (Tab’s)n (%)	Newlydevelopedthyroid dysfunction n (%)	Reference
1	USA	IFN α	237	NR	6 (2.5)	[25]
2	France	IFN α	68 (39/29)	4 (5.9)	8 (12)	[34]
3	Italy	IFN α	11241	NR	67 (0.6)	[6]
4	Japan	IFN α	677	NR	18 (2.7)	[31]
5	Japan	IFN α	439	NR	17 (3.9)	[80]
6	Japan	IFN α	109 (77/32)	2 (1.9)	9 (8.2)	[24]
7	Japan	IFN α	58 (37/21)	19 (32.8)	2 (3.4)	[42]
8	Italy	IFN α	114(79/35)	36 (31.5)	8 (7)	[22]
9	Italy	IFN α	120	NR	40 (33.3)	[12]
10	Italy	IFN α	75 (50/25)	26 (34.6)	5 (6.7)	[81]
11	Italy	RIFN 2α	78	31 (40)	27 (34.6)	[37]
12	Spain	IFN α	144 (95/49)	7 (4.9)	4 (2.8)	[82]
13	Italy	RIFN α	32 (26/6)	3 (9.3)	11 (34.4)	[9]
14	Italy	IFN α	114 (79/35)	36 (31.6)	12 (10.5)	[22]
15	Spain	IFN α	134	27 (20)	16 (12)	[30]
16	Norway	#IFNα	128	16 (6.5)	15 (11.7)#	[49]
	-do-	$IFN α	126	0	9 (11.9)$	[49]
17	China	IFN α	150	NR	28 (18.7)	[32]
18	Australia	IFN α	246	NR	9 (3.7)	[83]
19	Italy	Ribavirin	72 (25/47)	17 (23.6)	11 (15.3)	[27]
20	Italy	IFN α	75 (23/52)	17 (22.7)	3 (4)	[27]
21	Japan	IFN α	439(278/161)	NR	17 (3.9)	[36]
22	Italy	IFN α	136 (96/40)	64 (47)	16 (11.8)	[84]
23	Italy	IFN α	130	27 (21.1)	NR	[85]
24	France	IFN α	301	NR	30 (10)	[86]
25	Japan	IFN α	439	NR	17 (3.9)	[87]
26	Japan	IFN α	42	5 (12)	6 (14.3)	[45]
27	Canada	IFN α	54	NR	3 (5.5)	[88]
28	France	IFN α	12	NR	2 (16.7)	[89]
29	Japan	IFN α	50	NR	6 (12)	[90]
30	China	IFN α	88	NR	7(7.9)	[51]
31	Germany	IFN α	21 (12/9)	5 (23.8)	3 (14.3)	[71]
Overall frequency			16149	342 (20.6)+	432 (2.7)++	

Abbreviations and symbols in Table: RIBA, Ribavirin; IIT, Interferon induced thyroiditis; M/F, male/female ratio; NR, not reported; Tab, thyroid antibodies;

# IFNa 2b (total dose 366 MIU);

$ IFN alpha therapy (total dose 234 MIU);

+ frequency % calculated out of studies reporting Tab’s in 1656 patients and 16 studies;

++ frequency % calculated out of studies reporting thyroid dysfunctions in 16019 patients and 30 studies; RIFN, Recombinant IFN.

**Table 2:** Frequency of newly developed thyroid antibodies and clinical thyroid Disease (Including Autoimmune IIT and Non-Autoimmune IIT) in Patients with Hepatitis C Infection treated with combination therapy (IFN α (pegylated or non-pegylated+Ribavirin or Levovirin) treatment.

Si No	Country	Treatment	No.(M/F)	Newly developed thyroid antibody (Tab’s)n (%)	Newlydevelopedthyroid dysfunction n (%)	Reference
1	Italy	IFN-α 2b+RIBA	36	10 (27.8)	10 (27.7)	[10]
	-do-	CIFN α+RIBA	15	5 (33.3)	15 (100)	[10]
2	USA	IFN-α 2b+RIBA	225	NR	15 (6.7)	[41]
3	Germany	PEG-IFN α+RIBA	59	NR	11 (18.6)	[91]
4	Pakistan	IFN α-2b+RIBA	107	NR	20 (18.7)	[50]
5	Brazil	IFN α-2b+RIBA	65	4 (6.15)	3 (4.6)	[60]
6	China	IFN α+RIBA	161	NR	14 (8.69)	[51]
	-do-	Peg IFN α +RIBA	343	NR	47 (13.70)	[51]
7	UK	IFN α+RIBA	260 (172/88)	NR	58 (22.3)	[62]
8	Brazil	IFN α+RIBA	107	1(0.93)	5 (4.6)	[58]
9	Australia	IFN α 2b (IFN α)+RIBA	272	NR	18 (6.7)	[67]
10.	Pakistan	IFN α+RIBA	100 (77/23)	NR	18 (18)	[66]
11.	Germany	Peg IFN α 2b +RIBA	61*	7 (11.5)	6 (9.8)	[69]
12.	Taiwan	IFN α 2b	391	0	49 (84.8)	[70]
	-do-	Peg IFN α 2b +RIBA	70	0	9 (12.8)	[70]
13	Taiwan	IFN α	95	11 (11.6)	14 (14.7)	[79]
14	Germany	IFN α+RIBA	40 (19/21)	2 (5)	3 (7.5)	[71]
	-do-	Peg IFN α +RIBA	62 (29/33)	7 (11.3)	6 (9.6)	[71]
15	Poland	Peg IFN α +RIBA	30^**^	NR	2 (6.7)	[72]
16	Germany	Peg IFN α +RIBA or Levovirin	21	NR	0^***^	[73]
17	Australia	Peg IFN α +RIBA	18 (6/12)	1 (5.56)	0^a^	[75]
18	Australia	Peg IFN α +RIBA	11 (4/7)	0	0@	[7]
19	Greece	Peg IFN α +RIBA	61	NR	13 (21.3)	[64]
20	Australia	Peg IFN α+RIBA	511	NR	45 (8.8)	[65]
21	Turkey	Peg IFN α +RIBA	119 (21/98)	5 (25)	20 (16.8)	[57]
22	Korea	Peg IFN α +RIBA	1#	0@@	1 (100)	[58]
23	Greece	Peg IFN α+RIBA	109 (56/53)	5 (7)	26 (23.8)	[59]
24	Poland	IFN α+RIBA	89 (57/32)	7 (7.6)	12 (13.5)	[61]
25	Australia	Peg IFN α 2b +RIBA	3#(1/2)	NR	1 (33.4)	[63]
Overall frequency			3442	65 (5)^##^	441 (12.8)	

Abbreviations and symbols in Table: RIBA, Ribavirin; IIT, Interferon induced thyroiditis M/F, male/female ratio; NR, not reported; Tab: thyroid antibodies;

* Children and adolescent (2–17 yrs); ^**^Children between 8–19 years; ^***^Although remaining within the reference interval. TSH was reported as increasing during therapy in this study;

a although 6 cases showed thyrotropin outcome profile variation during treatment, all recovered at the end of the study;

@3 patients show initial higher TSH from the normal range, but all patients including 3 patients had normal thyroid functions at the end of 36 months;

@@Tabs were elevated at the time of therapy cessation;

# Case report; ^##^Out of 1292 patients and 13 studies.

### Mono-therapy of IFN

The frequency of newly developed Tab during IFN mono-treatment was in the range between 1.9–47% in 16 studies whereas the newly developed thyroid dysfunction ranged from 0.6–34.6% in 30 studies (Table 1). From 31 studies and out of a total of 16149 patients, the overall frequency of newly developed thyroid dysfunction during IFN treatment (mono-therapy) was 2.7% (Table 1). In one study, the frequency was not reported. From 16 studies and 1656 patients, the overall frequency of occurrence of newly developed Tab during IFN therapy was 20.6% (Table 1). In 15 studies, the frequency of thyroid antibodies was not reported.

### Combination Therapy for IFN

The frequency of newly developed Tab during IFN treatment in combination therapy was in the range between 0–33.3% in 13 studies whereas the newly developed thyroid dysfunction ranged from 0–100% in 25 studies (Table 2). From 25 studies and out of a total of 3442 patients, the overall frequency of newly developed thyroid dysfunction during IFN treatment (combination therapy) was 12.8% (Table 2). From 13 studies and 1292 patients, the overall frequency of occurrence of newly developed Tab during IFN therapy was 5% (Table 2). There are 3 studies with no newly developed thyroid dysfunction and 2 studies with no Tabs during combination treatment. In 12 studies, the frequency of thyroid antibodies was not reported.

### Country Wide Publications on Mono-therapy

The 31 publications (mono-therapy) included studies from Japan (8 studies), USA (1 studies), France (3 studies), Italy (12 studies), Spain (2 studies), Norway (1 study), China (1 study), Australia (1 study), Germany (1 study), Pakistan (1 study) and Canada (1 study). 13 studies from Italy showed frequency of the newly developed Tab and thyroid dysfunctions in the range of 9.3–47% and 4–34.6%, respectively. Eight studies from Japan showed the frequency in the range of 1.9–32.8% for newly developed thyroid antibody and 2.7–14.3% for thyroid dysfunctions. Studies from other countries (with 1–3 studies) also demonstrated similar wide variations in the frequency of newly developed thyroid antibody and thyroid dysfunctions (Table 1).

### Country Wide Publications on Combination Therapy

The 25 publications (combination therapy) included studies from USA (1 study), Italy (1 study), Brazil (2 studies),UK (1 study), China (1 study), Australia (5 studies), Taiwan (2 study), Germany (4 studies), Pakistan (2 studies), Poland (2 studies), Greece (2 studies), Korea (1 study) and Turkey (1 study). 5 studies from Australia showed frequency of the newly developed Tab and thyroid dysfunctions in the range of 0–5% and 0–33.4%, respectively. Four studies from Germany showed the frequency in the range of 5–11.5% for newly developed thyroid antibody and 7.5–18.6% for thyroid dysfunctions. Studies from other countries (with 1–3 studies) also demonstrated similar wide variations in the frequency of newly developed thyroid antibody and thyroid dysfunctions (Table 2).

### Treatment-specific Thyroid-related Side Effects in HCV Patients Undergoing Single or Combination IFN α Treatment

#### Single IFN α treatment

Intriguingly, neither the IFN α dosage nor the virological treatment response was found to be related to the incidence of thyroid dysfunction as per one report [21]. The prevalence of thyrotoxicosis in HCV patients treated with IFN α was reported by another study to be 2–3% of the treated patients [14]. Another study concluded that though positive thyroid antibodies with normal thyroid function tests were the most common findings in patients treated with IFN α, thyroid dysfunction was usually described in no more than 15% of all the treated patients [18]. An earlier study [22] conducted on patients undergoing IFN alpha therapy for chronic HCV and with no evidence of pre-existing thyroid disease did not report any thyroid autoantibodies after IFN treatment. As per one report, 15% of the patients treated with IFN α showed thyroid dysfunctions [10].

In contrast, the long-acting pegylated IFNα (PIFN) treatment had a lower incidence of thyroid-related side effects compared to non-pegylated IFNα [23], [5]. In patients treated with IFNα, hypothyroidism occurred in 2.4–19% of the patients, especially in those with pre-existing thyroid autoimmunity [24], [4]. The duration of IFN treatment was found to be related to the occurrence of thyroid dysfunction [25]. Another study reported that IFNα could induce both autoimmune and non-autoimmune thyroiditis [26]. Treatment of CIFN α alone showed 6.5% patients with newly developed thyroid antibodies and 11.9% patients with thyroid dysfunctions in a single study (Table 1). In HCV patients, therapy with IFN α and Consensus Interferon (CIFN), namely IFN α con-1 had higher cytotoxic effects on thyroid cells and a higher incidence of destructive thyroiditis than therapy with IFN α [10]. RIBA treatment alone resulted in 23.6% patients with new thyroid antibodies and 15.3% patients with thyroid dysfunctions (Table 1).

#### Combination treatment of IFN α (Pegylated or non-pegylated+RIBA or Levovirin

Patients treated with IFN α+RIBA have a relative risk of 4.3 for developing thyroid dysfunction [27]. Hypothyroidism was found to be more frequent in patients undergoing this treatment. The risk of developing thyroid autoimmunity after treatment of IFN+RIBA can be a consequence of enhancement of the Th1 immune response, which induces cell-mediated cytotoxicity [27].

Our study further brings to front the following findings observed in an earlier study:(i) the addition of RIBA to IFN α therapy for Chronic Hepatitis C (CHC) was associated with a higher risk of hypothyroidism, (ii) Patients without thyroid autoantibodies after treatment with IFN α alone were protected from the development of thyroid autoimmunity and/or dysfunction in a second course of antiviral treatment with IFN α+RIBA, (iii) the development of hypothyroidism in patients with thyroid autoantibodies undergoing treatment with IFN α+RIBA was significantly associated with the long-term remission of CHC [27].The result of the meta-analysis with only four studies and 1231 subjects showed high risk of thyroid dysfunction using Pegylated IFN (PIFN) compared to ribavirin in combination with IFN [17].This study further suggested that the pegylation of IFN, in combination with RBV, had no aggravating effect on thyroid diseases in the hepatitis C-afflicted population [17].

### Pre-Disposing Factors Causing Thyroid-related Side Effects in HCV Patients

As discussed earlier, a wide range in the prevalence of thyroid-related side effects was observed in the same study locations - for instance, in Italy and Japan (Table 1).This shows that there is the possibility of factors other than IFN playing a role. The other factors like pathophysiological factors, gender and ethnicity, genetic predisposition, HCV viral factors and environmental factors can also lead to thyroid dysfunctions during IFN treatment, which was explained in detail below.

#### Pathophysiological factors

Pre-existing thyroid autoimmunity can emerge as an important risk factor for developing thyroid dysfunction during IFN therapy. The presence of thyroid peroxidase antibodies (TPO-Ab) before treatment was identified as a risk factor for the incidence of thyroid disease in 60% of HCV patients receiving IFN α [28]. The relative risk of developing thyroid dysfunction, mainly hypothyroidism, was reported to betwo to 14 fold higher in patients with pre-existing positive TPO-Ab, as compared to patients with negative antibodies [9], [29].

#### Gender and ethnicity

Women were found to be more susceptible than men to develop IFN-related thyroid disease in some studies [3], [18], [30], [31], [32], [51]. These reports show a relative risk of three to seven folds higher for female compared to male. There are other reports which don’t claim any gender based relationship for IFN-related thyroid disease [24], [25], [33], [34], [35]. A higher prevalence of positive antithyroid antibodies (12.7%) and hypothyroidism (8.3%) were observed in female HCV patients undergoing IFN therapy, compared to only 1% positive antithyroid antibodies and no thyroid disease, after IFN treatment [49]. In a multivariate analysis, female gender and being of Asian origin were independent predictors of the development of biochemical thyroid dysfunction during IFNα treatment [49].

#### Genetic predisposition

A genetic predisposition to thyroid autoimmune disease is probably necessary for the development of thyroid disease in patients treated with IFN [33], [49]. The remarkable variation in the prevalence of IFN-related thyroid disease may also reflect variability in individual predisposition and genetic susceptibility to the disease [8].

#### HCV infection or viral factors itself as a pre-disposing factor

HCV infection in a patient can lead to development of thyroid autoimmune disease [30], [32], [35], [37]. Among patients infected with HCV, 20–42% show positive thyroid antibodies [30], [37]. In support of this hypothesis, some viral features like mixed HCV genotype infection and low HCV RNA levels are reportedly related to increased risk of developing thyroid disease [32]. HCV proteins show amino acid sequence homology with those of thyroid antigens [28], [35]. The presence of HCV particle within the thyroid cells may additionally contribute further damage to the thyroid gland (77). Therefore, HCV patients may carry a predisposition to autoimmune reactions through the mechanism of molecular mimicry [28].

However, a population-based study excluded a specific role of HCV infection in determining the development of thyroid disease [38]. In the absence of interferon treatment, the link between antithyroid autoantibodies, thyroid dysfunction and HCV infection is still debated [8].

#### Excess or deficiency of iodine

Epidemiological and clinical evidence suggest that iodine supplementation in an iodine-deficient population may precipitate the onset of thyroid autoimmunity [39]. The concomitant administration of pharmacological quantities of iodine to euthyroid patients treated with IFN α did not increase the frequency of thyroid dysfunction, especially hypothyroidism [40]. Destructive thyrotoxicosis was also correlated to low radioiodine uptake [14].

### Aftermath of IFN Withdrawal

Several studies have put forth contradictory results regarding the reversibility of the effect of IFN therapy on thyroid function after withdrawal of the treatment. As per one study, IFN alpha-related thyroid autoimmunity was not a completely reversible phenomenon because some patients developed chronic thyroiditis [22]. Another relevant observation of the study [22] was the coexistence of thyroglobulin antibodies (Tg-Ab) and TPO-Ab at the end of the treatment. This is a predictive factor for the presence of thyroid dysfunction, even if subclinical, many years after IFN withdrawal.

Autoimmune thyroiditis may not be reversible after IFN therapy [13], but a complete recovery of thyroid function within a few months of IFN withdrawal was also reported in earlier studies [34], [41]. Another report suggested that the treatment of HCV with IFN was safe in patients, since thyroid diseases are mostly reversible after treatment [41]. However, others have reported only a partial reversal of the thyroid dysfunction [29], [30], [42].

These contrasting results may be due to either the variable length of follow-up after IFN withdrawal or differences in the criteria used to define the recovery from thyroid disease [16]. Thyroid autoantibodies remain indefinitely positive in about 50% of patients with IFN-induced thyroid disease, whereas in others, circulating antibodies disappear after IFN withdrawal [41].

The uncertainty in the clinical management of patients developing IFN-induced thyroid disease may also be due to the variable expressions and different long-term outcomes of this side effect [16].

### Managing IFN-induced Thyroid Dysfunction in HCV Patients

Perhaps the true prevalence of thyrotoxicosis or hypothyroidism is much higher than that reported in literature [14], [24], [29], because it is often transient and has mild clinical manifestations [14]. Moreover, the symptoms of thyroid diseases (i.e., fatigue, myalgia, anxiety, depression, weight loss) may be easily mistaken for the side effects of IFN therapy *per se* [28].

Hence, the systematic screening of thyroid gland function and TPO-Ab titers in all patients with HCV - before, during and after IFN alpha therapy - should be recommended. Also, patients should be informed of the associated risk of thyroid dysfunction [30], [43], [44], [45]. To minimize the side effects of IFN treatment like hypothyroidism in the HCV patients it is required to screen the patient for thyroid-related diseases before the onset of the therapy [5], [46].

Considering the significant association between HCV infection and autoimmune thyroid diseases (AITD), the detection of TPO-Ab and TG-Ab in all HCV patients, independent of IFN therapy, is suggested [47]. Controlled studies on a large scale are needed to evaluate the role of HCV *per se*, and that of PEG-IFN and RIBA in the development of autoimmune thyroid diseases [48].

IFN therapy has shown to have direct toxic effect on thyroid cells, resulting in thyrocyte apoptosis, rupture of follicles and release of thyroid hormones [79]. These pathophysiologic events manifest themselves in the form of the bi-phasic thyroid response (0–18 months of treatment: testing will falsely reassure with normal thyroid tests, 18–25 months treatment: testing will detect hyperthyroidism and 25–42 weeks will indicate hyperthyroidism) that is so classical of this type of thyroiditis [74]. Hence the study [74] suggest the need for regular monthly thyroid testing to fully document and diagnose this prevalent and exclusive thyroid dysfunction in HCV patients.

### Clinical Practice Guidelines for HCV

The current standard approach of European Association for the Study of Liver (EASL) and well accepted standard of care for chronic hepatitis C is treatment with a combination of pegylated INF alpha plus ribavirin [52]. Two pegylated IFN-α molecules can be used in combination with ribavirin. They are pegylated IFN-α 2a and pegylated IFN-α2b [52]. The American Association for the Study of Liver Diseases (AASLD) also proposes the recommended therapy of chronic HCV infection as the combination of a pegylated interferon alpha and ribavirin [53]. The choice of the regimen for pegylated interferon alpha and ribavirin was based upon the results of three pivotal, randomized, clinical trials that demonstrated the superiority of this combination treatment over standard interferon alpha and ribavirin [54]–[56]. Even though the clinical practice guidelines are mostly followed, the mono-therapy is still continued as treatment regimen for HCV patient as per the available literature in the present systematic review. There are recent studies with single therapy of IFN due to country specific treatment modalities following other than EASL and AASLD criteria.

### Constraints in Pooled Analysis of Studies

The wide variation among the frequency of side effects was observed in both single and combination therapy studies. The different dosage and treatment schedule and measurements of thyroid parameters at different time intervals viz., 3 months [49], [70], 2–3 months [76], 24 weeks [32] in the publications result in constraints for the outcome of the pooled analysis. Variations in definition/criteria for thyroid dysfunction followed in each study [Table 3] influence the outcome of the pooled risk estimate of side effects of thyroid dysfunction. Hence the overall frequency of thyroid dysfunctions and newly developed Tabs reported as side effects of mono and combination therapy of IFN in HCV patients from different studies in this systematic review analysis may have limitations of factors mentioned above.

**Table 3:** Differences in definition of thyroid dysfunction/positive for thyroid autoantibody given in method section of some of the publications.

References	Definition given in method section
[17], [67]	Thyroid dysfunction (TD) was defined as having hypo- orhyper-thyroidism, (clinically and/or biochemically based). Thyrotoxicosis was defined as having TSH <0.1 mU/L, either fT4 level >26.0 and/or FT_3_ level >5.5 pmol/l, respectively. Hypothyroidism was defined as having TSH level >4.0 mIU/L, withnormal or low (<10.0 pmol/L) fT4 levels.
[62]	Patients developing TD were classified as either hyperthyroid orhypothyroid on the basis of their first serum TSH abnormality.Patients with a serum TSH <0.27 mU/L were classified as hyperthyroid.Patients with hyperthyroidism identified with a serum TSHsuppressed to <0.01 mU/l were subject to a diagnostic thyroid isotope scan to identify those with Graves’ disease. All patients with hyperthyroidism not developing Graves’ disease were classified as having a transient thyroiditis [associated with transient, overt hyperthyroidism (free T4>22.0 pmol/L and/or free triiodothyronine (T3) >6.8 pmol/L) or a transient subclinical hyperthyroidism (free T4 and free T3 in the normal range)].Patients with hypothyroidism (serum TSH >4.2 mU/L) were categorized according to whether hypothyroidism was transient (acute or subclinical) or permanent requiring long-term levothyroxine replacement therapy following consultation with a specialist endocrinologist at completion of IFN/RBV as described later.
[60]	TSH (ultrasensitive third-generation method with a reference normal range of 0.35–5.50 mcIU/L), and FT_4_ (reference normal range of 0.58–1.40 ng/dL) were assayed using commercially available kits by immunometric assays.TPO-Ab was detected by solid phase 2-site sequential chemiluminescent immunometric assay (normal:<40.0 IU/mL).Patients were classified as positive (TPO-Ab >40.0 IU/mL) or negative (TPO-Ab <40.0 IU/mL) for thyroid autoimmunity
[69]	TSH elevated
[70]	Thyroid dysfunction (TSH <0.1 or >5 mU/L)
[71]	Along with testing auto-antibodies, thyroid function was evaluated by measuring the serum levels of free triiodothyronine (FT_3_; normal values: 1.8–4.6 ng/L), free thyroxine (FT_4_; normal values: 0.9–1.7 ng/dL) and thyroid-stimulating hormone (TSH; normal values:0.3–4.2 mU/L).Determined the anti-thyroglobulin antibody (anti-thyroid peroxidase antibodies (TPO; normal values: <35 IU/mL) in the samples.
[7]	Hyper- and Hypo-thyroidism:Thyrotoxicosis was defined as having TSH <0.1 mU/L, either FT_4_ level >26.0 and/or FT_3_ level >5.5 pmol/L, respectively. Hypothyroidism was defined as having TSH level >4.0 mIU/L, withnormal or low (<10.0 pmol/l) fT4 levels.
[59]	TD was assessed by the serum levels of free-thyroxine (FT_4_) and TSH. Thyroid autoimmunity was defined by elevated antithyrogobulin (TgAb) and antithyroperoxidase antibodies (TPOAb) (normal levels <60 IU/L).
[22], [27]	Patients with serum positivity for at least one thyroid autoantibody were defined as Abs +ive (TgAb>100U/ml and/or TPOAb >10U/ml).Overt hypothyroidism was defined by serum TSH values above normal range, serum FT_4_ below the normal range and serum FT_3_ in or below the normal range. The normal values were 3.8–7.7 pmol/L for FT_3,_9.0–23.1 pmol/L for FT_4_ and 0.3–3.5 mu/L for TSH.
[49]	Thyroid dysfunction was diagnosed when the TSH level was either >4.5 (hypothyroidism) or <0.2 MIU L^−1^ (hyperthyroidism). The diagnosis of symptomatic thyroid disease was based on the clinical judgment of the treating physician.
[92]	The criteria of diagnosing hyperthyroidism were, apart from the typical clinical symptoms, a decrease in TSH level <0.4 mIU/ml (normal 0.4–4.0 mIU/ml) and an increase of FT_4_ (normal range:0.8–1.9 ng/dl) and/or FT_3_ (normal range: 1.8–4.2 pg/ml).Subclinical hyperthyroidism was diagnosed in case of a decrease in TSH level and normal concentration of free thyroid hormones (TH).Hypothyroidism was diagnosed when the increased serum concentration of TSH and a decreased level of FT_4_ concentration were revealed. In a case of increased level of TSH within the limits of 5–10 mIU/ml and normal FT4 serum level,a latent hypothyroidism was diagnosed. Autoimmune thyroiditis (ATI) was diagnosed if an increased TPOAb level (normal range: 0–35 IU/ml) and/or TgAb (normal: 0–40 IU/ml) were found. Increased concentrations of TPOAb and/or TgAb (level >100 IU/ml) were set as a criterion of ATI diagnosis.
[64]	Thyroid dysfunction was defined as TSH level of either more than 4.0 (hypothyroidism) or less than 0.3 (hyperthyroidism) mU/L, irrespective of FT_3_/FT_4_ levels

### Conclusion

To conclude, the present review shows that there is a wide range in the incidence of newly developed thyroid dysfunctions and thyroid antibodies in IFN-treated HCV patients. IFN α therapy alone or in combination with other drugs has different effects on the incidence of thyroid dysfunctions. Several factors that pre-dispose an HCV patient to acquire thyroid related abnormalities during IFN treatment have been discussed. These may include gender and ethnicity, HCV viral factors, genetic predisposition, and environmental and patho-physiological factors among others. Variations in IFN dosage, treatment duration of IFN, definition/criteria followed in each study for thyroid dysfunction and irregular thyroid function testing during treatment in different studies influence the outcome of each study and render the pooled risk analysis of side effects of thyroid dysfunction difficult. Another aspect highlighted by this systematic review is the variability that occurs among reports discussing the reversibility of thyroid dysfunction after IFN withdrawal. This is a comprehensive attempt to collate relevant data from 56 publications across several nations about IFN (both mono and combination therapy) related thyroid dysfunction among HCV patients. The role of each factor in causing thyroid dysfunctions in HCV patients treated with IFN should be analyzed in detail in future studies, for a better understanding of the problem and sounder clinical management of the disease.
